# Smad7 ameliorate small airway remodeling in COPD by modulating epithelial-mesenchymal transition

**DOI:** 10.18332/tid/210414

**Published:** 2025-10-31

**Authors:** Xianyang Liu, Shenghua Sun, Shengyang He, Lihua Xie

**Affiliations:** 1 Department of Pulmonary and Critical Care Medicine, The Third Xiangya Hospital of Central South University, Changsha, China; 2 Department of Pulmonary and Critical Care Medicine, The Second Xiangya Hospital of Central South University, Changsha, China; 3 Research Unit of Respiratory Disease, Central South University, Changsha, China; 4 Clinical Medical Research Center for Pulmonary and Critical Care Medicine, Changsha, China; 5 Diagnosis and Treatment Center of Respiratory Disease, Central South University, Changsha, China

**Keywords:** Smad7, small airway remodeling, COPD, EMT

## Abstract

**INTRODUCTION:**

Small airway remodeling is a key pathological feature of COPD, yet its mechanisms remain unclear. TGF-β1 induces epithelial-mesenchymal transition (EMT), contributing to airway remodeling. Smad7 is a negative regulator of TGF-β signaling, but its role in COPD remains undefined. This study investigates whether Smad7 suppresses TGF-β1-induced EMT in COPD small airway remodeling.

**METHODS:**

Lung tissues from COPD patients (n=3 for each group) and mouse models (n=5 for each group) were analyzed for EMT markers and collagen deposition. BEAS-2B cells were exposed to cigarette smoke extract (CSE) to assess TGF-β1 secretion. EMT markers (E-Cadherin, N-Cadherin, and Vimentin) were evaluated using RT-qPCR, Western blot, and immunofluorescence staining. Morphological changes were examined, and Smad7 function was assessed via overexpression and knockdown experiments.

**RESULTS:**

COPD patients and mouse models showed increased EMT and collagen deposition. CSE exposure upregulated TGF-β1 in BEAS-2B cells, leading to decreased E-Cadherin and increased N-Cadherin and Vimentin. Morphological changes confirmed EMT induction. Overexpression of Smad7 reversed these effects, while its knockdown enhanced them.

**CONCLUSIONS:**

Smoking promotes TGF-β1-induced small airway remodeling in COPD by driving EMT. Smad7 suppresses this process.

## INTRODUCTION

Chronic obstructive pulmonary disease (COPD) is one of the leading causes the of morbidity and mortality around the world. In China, the estimated COPD population aged >20 years is 99.9 million, with the overall prevalence rate of 13.7% age >40 years^1^, which makes it the third most common chronic disease after hypertension and diabetes mellitus, bringing about heavy social and economic burden, and serious damage to human health^2^.

COPD is characterized by persistent respiratory symptoms and airflow limitation^3^, the most important pathophysiological basis of which is small airway remodeling^4^. Our previous studies have confirmed the presence of small airway lesions such as airway mucosa thickening, massive inflammatory cells infiltration and small airway narrowing in early stage of animal models exposed to cigarette smoke^5^. The aspects of small airway remodeling are epithelial mucous metaplasia, lung parenchyma destruction, collagen deposition and fibrosis of airway wall etc., of which the mechanisms remain largely unknown^6^. At present, the mainstay drug therapies for COPD are bronchodilators, inhaled corticosteroids, etc.^7^, whose suppressive effect on airway remodeling are not yet confirmed^8^.

According to Milara et al.^9^, smoking-related changes of epithelial-mesenchymal transition (EMT) were found in the small airways of COPD patients, as well as an increased expression of S100A4 and Vimentin in lung tissue^10^. Meanwhile the reticular basement membrane fragmentation, correlated with decreased lung function, suggested that the structural changes of small airways are closely related to EMT in COPD patients. EMT refers to the complete or partial transformation of epithelial cells into mesenchymal cells, which plays an important role in tissue repair, organ fibrosis and tumorigenesis^11^. A previous X-Gal staining found that approximately one-third of S100A4-positive fibroblasts in a pulmonary fibrosis mouse model were derived from epithelial cells through EMT^12^. Thus, we speculate that EMT plays an important role in the small airway remodeling of COPD. Yet the specific regulatory mechanism of EMT remains poorly understood and needs to be further studied.

Among all the molecules that induce EMT, TGF-β is the one of greatest interest^13^. By acting through the downstream Smad signaling pathway, it can directly induce EMT between cells in culture and play a key role in tumor-associated EMT. It has been reported that TGF-β1/Smad makes an important activating pathway of EMT in prostatic hyperplasia and esophageal carcinoma^14^. Consistent with this, our study found that there is abnormal expression of TGF-β/Smad7 in lung tissue of COPD patients^15^, which suggests that Smads/TGF-β pathway may play an important part in the pathogenesis of COPD.

## METHODS

### Collection of human lung tissues

Lung tissue samples were collected from both COPD patients (demographic data of enrolled COPD patients are shown in Table 1) and healthy controls undergoing pulmonary wedge resection for lung cancer or lung bullae, at the Third Xiangya Hospital of Central South University in Changsha, China (from 1 August 2017 to 1 January 2019). All tissue samples were collected at least 2 cm away from the primary focus (the primary lesion of the original disease). Following collection, the tissues were immediately frozen in liquid nitrogen and stored at -80°C for subsequent analysis. COPD diagnosis was based on GOLD 2017 criteria^3^, with patients presenting with other structural lung diseases such as pulmonary fibrosis, tuberculosis, or bronchiectasis being excluded from the study. Verbal consent was obtained from each participant, as this study would not give any additional harm or surgical procedure to them. Participants discussed the study details with a researcher via telephone or video call. Their consent information was recorded in the participant contact form for this study, with another researcher witnessing the entire process and signing the contact form to confirm. Ethical approval for this study and all experimental protocols were approved by the Institutional Review Board of the Third Xiangya Hospital of Central South University (Approval No. 2017-S072), and the study is reported in accordance with ARRIVE guidelines (https://arriveguidelines.org). Patients or the public were not involved in the design, or conduct, or reporting, or dissemination plans of our research.

**Table 1:** Demographic data of enrolled COPD patients

	*COPD patients* *(N=3)* *Mean ± SE*	*Healthy people* *(N=3)* *Mean ± SE*	p*
Age (years)	58.7 ± 11.0	67.8 ± 9.2	<0.05
Males/Females	3/0	3/0	-
FEV1/FVC	53.15 ± 8.92	80.99 ± 5.5	<0.05
FEV1/FEV1-predicted	65.03 ± 9.47	112.60 ± 11.21	<0.05
Smoking/No smoking	3/0	0/3	-

* Independent-sample t-test. SE: standard error.

### Preparation of cigarette smoke extract (CSE)

The preparation of cigarette smoke extract (CSE) followed the procedure outlined in our previous study^16^. In brief, a non-filter Furong cigarette (tar 13 mg, nicotine 1 mg, CO 14 mg, China Tobacco Hunan Industrial Co. Ltd, Changsha, Hunan, China) was lighted, and the resulting smoke was dissolved in 4 mL of phosphate-buffered saline (PBS) using a constant-pressure vacuum pump set to -0.1 kPa. Subsequently, particles and bacteria were removed from the CSE solution by passing it through a 0.22 μm micropore filter (Millipore, Billerica, MA, USA). The filtered CSE solution (100% concentration) was then utilized for further experiments within 30 minutes of preparation. For the *in vitro* experiment, we selected 5% of the filtered CSE solution for 24-h exposure in BEAS-2B cells.

### COPD animal model

The COPD mouse model was induced by combining cigarette smoke exposure and intraperitoneal injection of cigarette smoke extract (CSE), as previously described^17^. Ten male C57BL/6 wild-type mice (aged 6–8 weeks) were obtained from Hunan SJA Laboratory Animal Co. Ltd (Changsha, Hunan, China). They were randomly divided into COPD model (n=5) and control (n=5) groups. All the containers of mice and living conditions remain the same. Mice in the COPD model group were exposed to cigarette smoke twice daily for 28 days (mice were given CS exposure in a sealed box with a ventilation hole for two cycles per day and 8 cigarettes for 15 min per cycle), excluding days 1, 12, and 23. Additionally, they received intraperitoneal injections of 100% CSE solution (0.3 mL/20 g) on days 1, 12, and 23. Control group mice were exposed to fresh air and received equivalent volumes of phosphate-buffered saline (PBS) injections. Exclusion criteria of enrolled mouse: 1) the mouse dies unexpectedly during the modeling process or after model establishment, making it impossible to complete the experiment; 2) the mouse develops severe health issues unrelated to the model (e.g. severe infections, fractures, extreme weakness); 3) unanticipated behaviors or physiological changes that interfere with experimental interventions or outcome observations (e.g. sustained abnormal weight loss or loss of appetite); 4) procedural errors lead to improper modeling (e.g. incorrect injection site, inaccurate dosing, or mishandling); 5) the mouse is mistakenly assigned to the wrong group or experimental procedure; and 6) the mouse exhibits significant pain or distress, reaching the termination criteria specified by the animal ethics committee. On day 28, all mice were euthanized (before euthanasia, mice were administered intraperitoneal anesthesia with ketamine, 50 mg/kg), and their right lung tissues were collected and fixed in 4% paraformaldehyde. Subsequent histological analyses included hematoxylin and eosin (H&E) staining (both human lungs and mice lungs, in paraffin-embedded sections), Masson staining (both human lungs and mice lungs, in paraffin-embedded sections), and immunohistochemical analysis (all methods were carried out in accordance with relevant guidelines and regulations). Only the first author knew the grouping strategy and the location of the present study.

### H&E staining

Tissues of both human and mice lungs were inflated by 4% paraformaldehyde at a constant pressure of 25cmH2O and then fixed with 4% paraformaldehyde for 24 h. The fixed lung tissue was embedded by paraffin (Sigma-Aldrich Co., St Louis, MO, USA) and sectioned into 4-μm sections for further staining with H&E (Sigma-Aldrich Co.). After deparaffinization and rehydration of the slides, they were immersed in hematoxylin solution for 10 min followed by rinse for hematoxylin staining. Then, the slides were immersed in eosin Y solution for 2 min for eosin staining.

### Immunohistochemical staining

Following the dewaxing and hydration steps, tissue sections (both human lungs and mice lungs, in paraffin-embedded sections) underwent antigen retrieval by heating PBS pH 9.0 for 25 min. Subsequently, endogenous enzyme interference was eliminated, and the sections were then incubated overnight at 4°C with primary antibodies against E-Cadherin (1:100, Proteintech, Cat No: 20874-1-AP) or N-Cadherin (1:100, Proteintech, Cat No: 22018-1-AP). Following this, the sections were treated with the corresponding enzyme-labelled secondary antibody (Zhongshan Goldenbridge Biotechnology Co. Ltd., Beijing, China) for 30 min at room temperature. Visualization of the target proteins was achieved using Diaminobenzidine (Zhongshan Goldenbridge Biotechnology Co. Ltd., Beijing, China). The absorbance optical density (AOD) of positive area (only bronchial epithelial cells) of lung tissue was quantified using Image J software (Version 1.53v).

### ELISA method

The ELISA reagent kit (Elabscience, Wuhan, China, Cat No: E-EL-0162c) was enrolled in this method according to the user manual. Briefly, supernatant from cells was collected, centrifuged at 1000 g for 20 min, and the pellet was discarded. To 20 μL of sample dilution buffer, 100 μL of supernatant was added, followed by 40 μL of activation reagent 1. After 10 min at room temperature, 40 μL of activation reagent 2 was added. The standard was centrifuged at 10000 g for 1 min, dissolved in 1.0 mL dilution buffer (10 ng/mL), and serially diluted to prepare a standard curve. Samples and standards (100 μL) were added to an ELISA plate and incubated at 37°C for 90 min. After washing, 100 μL of biotinylated antibody was added and incubated for 1 h. The plate was washed, and 100 μL of HRP enzyme was added and incubated for 30 min. After five washes, 90 μL of TMB was added and incubated for 15 min. The reaction was stopped, and the OD was measured at 450 nm.

### siRNAs and plasmids

Smad7 siRNAs and a negative control were designed and synthesized by Guangzhou RiboBio Co., Ltd (Guangzhou, Guangdong, China). Smad7 overexpression plasmid was constructed by Shandong Vigene Biosciences Co., Ltd (Jinan, Shandong, China). on the basis of pENTER vector.

### Cell transfection

BEAS-2B cells in exponential growth phase were seeded at a density of 2×10^5^ cells/well in 6-well plates and cultured overnight to 50–60% confluency. On the day of transfection, cells were switched to serum-free medium. For knockdown experiments, 20 nM siRNA was transfected using Lipofectamine 3000 (Invitrogen, Carlsbad, CA, USA) at a 1:3 nucleic acid-to-reagent ratio. For overexpression, 2 μg plasmid DNA was transfected under the same conditions. After 6 h, the medium was replaced with fresh complete medium, and cells were harvested 48 h post-siRNA transfection or 24–48 h post-plasmid transfection for downstream analyses. Transfection efficiency was confirmed by qPCR and Western blot analysis of SMAD7 expression.

### RT-qPCR

Total RNA of cells was extracted using Trizol reagent (Invitrogen, Carlsbad, CA, USA) following manufacturer’s protocol, and cDNA was synthesized using reverse transcription system (Promega, Madison, WI, USA). Target mRNAs were amplified using the GoTaq qPCR Master Mix (Promega, Madison, WI, USA) and relative expression was detected by a real-time PCR system (Applied Biosystems, Foster City, CA, USA). The primers were designed and synthesized by Sangon Biotech (Shanghai) Co., Ltd. of which sequences are shown in Table 2.

**Table 2:** Primers sequence

*Gene*	*Primers sequence*
SMAD7	F: CTCGGAAGTCAAGAGGCTGTGTTG
	R: TCTAGTTCGCAGAGTCGGCTAAGG
E-Cadherin	F: GCTCTTCCAGGAACCTCTGTGATG
	R: AAGCGATGGCGGCATTGTAGG
N-Cadherin	F: AAGGTGGATGAAGATGGCATGGTG
	R: TGCTGACTCCTTCACTGACTCCTC
a-SMA	F: TTCGTGACTACTGCTGAGCG
	R: CTGTCAGCAATGCCTGGGTA
Vimentin	F: TTCGTGAATACCAAGACCTG
	R: ATCCTGCTCTCCTCGCCTTC
GAPDH	F: ACAGCCTCAAGATCATCAGC
	R: GGTCATGAGTCCTTCCACGAT

### Western blot

Total protein was extracted using RIPA Lysis Buffer Strong (Beyotime, Shanghai, China) and protein was quantified by Bicinchoninic acid assay (Thermo Scientific, Rockford, lL, USA) method. The specific antibodies used in the study were E-Cadherin (1:1000, Proteintech, Cat No: 20874-1-AP), N-Cadherin (1:1000, Proteintech, Cat No: 22018-1-AP), Vimentin (1:2000, Proteintech, Cat No: 10366-1-AP), GAPDH (1:5000, Proteintech, Cat No: 60004-1-Ig), Smad7 (1:1000, Proteintech, Cat No: 25840-1-AP), and the secondary anti-rabbit antibody (Proteintech, Rosemont, IL, USA) was used to develop the bands, and the gray-scale values were used to analyze the relative expression of the target protein.

### Statistical analysis

Measurement data are reported as mean ± standard error. The differences among groups were analyzed by one-way ANOVA, while comparisons between two groups were analyzed by independent-sample t-tests. Prior to one-way ANOVA, the normality of the dependent variables was tested using the Shapiro-Wilk test, and homogeneity of variance was assessed with Levene’s test. For datasets meeting these assumptions, pairwise comparisons were performed using Tukey’s *post hoc* test with correction for multiple comparisons. Given the small sample size, permutation tests (10000 permutations) were additionally performed for each comparison to obtain distribution-free p-values, thereby reducing the influence of distributional assumptions. When the normality assumption was violated, non-parametric tests were applied instead: the Mann-Whitney U test for two-group comparisons, and the Kruskal-Wallis test followed by Dunn’s *post hoc* test with Bonferroni correction for multiple comparisons for multi-group analyses. A difference was considered statistically significant when p<0.05. All data were analyzed using GraphPad Prism6.0 and SPSS 18.0.

## RESULTS

### Airway remodeling and EMT in lungs of COPD patients and mice models

**Figure 1:**
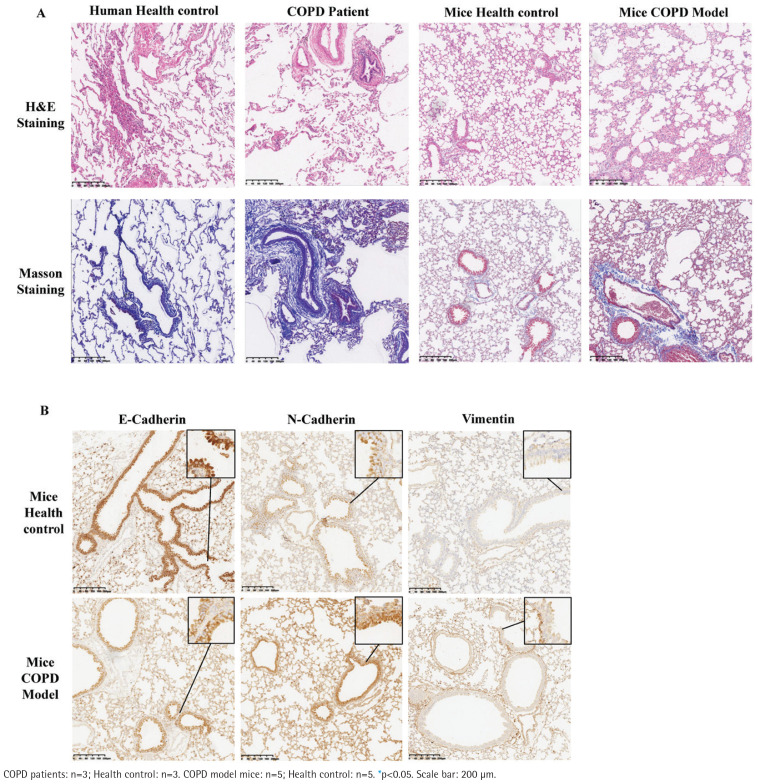
Airway remodeling and EMT in lungs of COPD patients and mice models. H&E and Masson staining of lungs from different group indicating, in both COPD patients and COPD model mice, massive inflammatory cell infiltration in the lung parenchyma, alveolar septum rupture, expansion of the alveoli, and partly thickening of the fused alveolar septum, more collagen deposition around small airways could all be observed (A). The following Immunohistochemical staining revealed higher E-Cadherin and lower N-Cadherin and Vimentin in small airway epithelial cells of COPD model mice (B and C). Further Western blot of lungs from COPD patients and health people reconfirmed the immunohistochemical staining results (D)

The H&E staining in both COPD patients and the COPD mouse model group revealed massive cellular infiltration in the lung parenchyma, alveolar septum rupture, expansion of the alveoli, and partly thickening of the fused alveolar septum (Figure 1A). Masson’s staining showed increased deposition of collagen fibers in the airway wall in both COPD model group and COPD patients compared with the control group (Figure 1A), suggesting an increase in the extent of airway fibrosis and the presence of airway remodeling in the COPD group mice.

**Figure 2:**
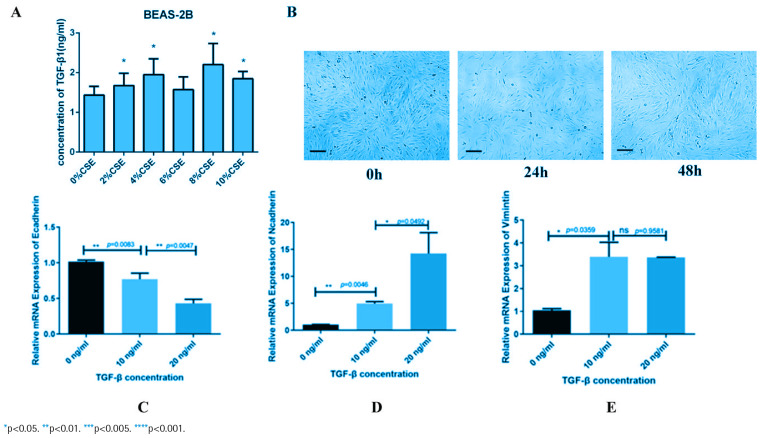
CSE enhance EMT process of bronchial epithelial cells through TGF-β1. Different CSE exposure on BEAS-2B cells lead to increased TGF-β1 in supernatant in a concentration dependent manner, confirmed by ELISA test (A). Shape of BEAS-2B cells changed into spindle-shaped changes, exhibiting a spindle-like appearance, with a significant reduction in intercellular connections (B). Extra exposure of TGF-β on BEAS-2B cells lead to lower mRNA level of E-Cadherin (C) and higher (D) and Vimentin (E), tested by rt-qPCR. Further Western blot (F-G) and immunofluorescent staining (H-I) reconfirmed the rt-qPCR results

Despite the pathological changes in lungs, we verified through both immunohistochemical (IHC) staining (Figure 2B; and Supplementary file Figure 2A) and Western blot (WB) analysis (Supplementary file Figure 2B) that the expression of the epithelial marker E-Cadherin decreased, while the mesenchymal markers N-Cadherin and Vimentin increased in the group of mice with COPD model. These alterations were predominantly observed in the bronchial epithelial cell (Figure 2B).

### CSE enhance EMT process of bronchial epithelial cells through TGF-β1

Exposure to cigarette smoke extract (CSE) led to an upregulation of TGF-β1 secretion in bronchial epithelial cells BEAS-2B (Figure 2A). Under the stimulation of TGF-β1, cells undergo spindle-shaped changes, exhibiting a spindle-like appearance, with a significant reduction in intercellular connections (Figure 2B). Further, the mRNA level of E-Cadherin was descendant and N-Cadherin, Vimentin was ascendant in a concentration dependent manner (Figures 2C–2E). Meanwhile, E-Cadherin and Vimentin also exhibit similar changes at the protein level, confirmed by Western blot (Supplementary file Figures 2C and 2D) and immunofluorescence staining (Supplementary file Figures 2E–2H).

### Smad7 modulates TGF-β1 induced EMT in bronchial epithelial cells

Transfection with a Smad7 overexpression plasmid partially reversed the EMT process induced by TGF-β1 in BEAS-2B cells. Specifically, E-Cadherin increased, while N-Cadherin and Vimentin decreased, as confirmed by Western blot analysis (Supplementary file Figures 1A and 1B). In contrast, transfection with siRNA targeting Smad7 resulted in reversed results at the mRNA level. However, Vimentin has no differences between groups (S2C). TGF-β1 stimulation not only had no effect on the efficacy brought by siRNA targeting Smad7 (Supplementary file Figure 1D). At the protein level, as confirmed by Western Blot analysis, Smad7 overexpression partly blocked the TGF-β1-induced EMT, while siRNA targeting Smad7 enhanced this phenomenon (Supplementary file Figures 1E–1G).

## DISCUSSION

COPD is characterized by partially reversible airflow limitation, with airway remodeling being a common pathological mechanism. Research suggests that injury to the airway epithelium contributes to this remodeling, highlighting the significant role played by airway epithelial cells. However, the precise mechanisms involved remain unclear. Our study reveals evidence of EMT in both COPD patients and animal models, with the TGF-β1/Smad7 pathway identified as a regulator of this process.

Research by Gu et al.^4^ demonstrated that exposure to cigarette smoke led to increased collagen deposition and α-SMA expression in human bronchial epithelial (HBE) cells and mouse lungs exposed to cigarette smoke. Additionally, significant alterations in EMT biomarkers were observed in COPD patients, including increased levels of N-Cadherin and Vimentin, and decreased levels of E-Cadherin^18^, suggesting the involvement of EMT in small airway lesions in COPD^19^. Our own investigations corroborate these findings, revealing airway remodeling and collagen deposition in both COPD patients and animal models, accompanied by the presence of EMT biomarkers. Furthermore, our studies demonstrate that cigarette smoke extract (CSE) can induce EMT in human bronchial epithelial cells. Collectively, these results underscore the potential role of EMT in airway remodeling in COPD.

Previous research has identified multiple signaling pathways that regulate different types of EMT, including the Wnt/β-catenin pathway, TGF-β1/Smad pathway, MAPK/P38 pathway, PI3K/Akt pathway, and NF-κB/Twist pathway. Notably, EMT mediated by the TGF-β1/Smad pathway has been implicated in the pathogenesis of various conditions such as posterior capsular opacification^20^, hepatic fibrosis^21^, and diabetic nephropathy^22^. Despite this, limited attention has been given to the role of TGF-β1/Smad in COPD, with only a few studies exploring its inhibitory effects using compounds such as Silibinin^23^ and Ginsenoside^14^.

In our previous study^24^, we observed abnormal expression of TGF-β1/Smad pathway biomarkers in COPD patients and animal models. However, no investigation has explored the relationship between TGF-β1/Smad and COPD-related EMT. In this study, we discovered that cigarette smoke exposure increases the expression of TGF-β1. These findings lay a solid foundation for further elucidating the regulatory mechanisms of the TGF-β1/Smad signaling pathway.

Previous research conducted by Mahmood et al.^25^ has indicated that levels of activated Smad 2, 3 (receptor-activated), and 7 (inhibitory) proteins may correlate with EMT markers and changes in lung function. It is widely accepted that Smads-mediated EMT involves the translocation of phosphorylated Smad complexes into the nucleus, leading to the synergistic upregulation of extracellular matrix gene expression alongside other transcription factor^26^. However, the precise mechanisms underlying this process require further investigation. Smad7, a crucial inhibitor of the TGF-β pathway, plays a significant role in regulating the expression of EMT markers^27^. Our study found that overexpression of Smad7 inhibited TGF-β1-induced EMT, while conversely, inhibition of Smad7 significantly enhanced TGF-β-induced EMT. These findings suggest that Smad7 may play a pivotal role in TGF-β-induced EMT.

EMT is also a key mechanism in pulmonary fibrosis, another condition frequently observed in conjunction with COPD. Therefore, studying the relationship between EMT and pulmonary fibrosis has clinical significance.

### Limitations

Several limitations should be acknowledged. First, the cross-sectional nature of this study allows only the identification of associations rather than causal relationships. Second, functional validation of the observed EMT-related changes was not performed, and in vivo experiments were lacking. Third, the relatively small sample sizes for both COPD patients and animals may increase the risk of bias. In addition, the potential influence of other regulatory proteins and transcription factors in the TGF-β1/Smad pathway on EMT, as well as the link between EMT and airway remodeling, remains unclear. Future studies with larger sample sizes, functional assays, and animal models will be necessary to address these limitations and further clarify the role of Smad7 in COPD.

## CONCLUSIONS

This study confirms the presence of airway remodeling and EMT in both COPD patients and animal models. Cell experiments revealed that EMT induction by TGF-β1 is concentration- and time-dependent, while identifying Smad7 as a key negative regulator of EMT.

## Data Availability

The data supporting this research are available from the authors on reasonable request.
